# Calculation model and experimental study of the collapse strength of titanium alloy tubing and casing

**DOI:** 10.1038/s41598-022-08636-9

**Published:** 2022-03-16

**Authors:** Qiang Liu, Ning Li, Zhao-xi Shen, Mi-feng Zhao, Jun-feng Xie, Guo-chuan Zhu, Xin Xu, Cheng-xian Yin

**Affiliations:** 1 State Key Laboratory for Performance and Structural Safety of Petroleum Tubular Goods and Equipment Materials, CNPC Tubular Goods Research Institute, No. 89, Jinye 2nd Road, Xi’an, 710077 Shaanxi China; 2 Petroleum Engineering Institute, Tarim Oil Field Company of CNPC, Kuerle, 841000 Xinjiang China

**Keywords:** Materials science, Structural materials

## Abstract

Titanium alloy has become a promising candidate material for oil country tubular goods (OCTGs) in harsh service environments owing to its high specific strength, low density, low elastic modulus, excellent toughness, excellent anti-fatigue and corrosion resistance. However, because the high-quality natural gas resources in China are mainly concentrated deep underground, titanium alloy tubing and casing will bear great external pressure loads underground, so the collapse strength of titanium alloy tubing and casing is very important for the safety of the string in the well. In this paper, a new collapse strength calculation model, the strength collapse criterion model (SCM), was proposed for titanium alloy tubing and casing. 35 different specifications of titanium alloy tubing and casing were selected for the full-scale collapse tests to verify the reliability of the established SCM model. Furthermore, the effect of different key parameters (such as strength, ovality, eccentricity and residual stress) on collapse strength of titanium alloy pipes were investigated systematically and compared with the same specifications of steel pipes. The strength collapse criterion model and analysis results can provide a technical reference for the design and use of titanium alloy OCTGs in the petroleum and natural gas industries.

## Introduction

With the development of unconventional oil and gas resources in deep and ultra-deep well of China^1,2^, the requirements for high-performance oil country tubular goods (OCTGs) are increasing. As the main pipe string channel and protective shield for deep oil and gas development, the tubing and casing not only bear the extremely combined load of the high-temperature and high-pressure in the well, but also bear the corrosion effects of hydrogen sulfide, carbon dioxide, a high concentration of chloride ion and even elemental sulfur in service conditions^3,4^. Once a failure accident occurs, it will not only cause the failure of the deep energy exploration and bring considerable economic loss, but also pose a threat to safety and environmental damage. Therefore, OCTGs are extremely important to the oil and gas industry. Titanium alloy materials have become a hot research direction for oil country tubular goods (OCTGs) and offshore components in harsh service environments owing to their high specific strength, low density, low elastic modulus, excellent toughness, and excellent fatigue and corrosion resistance^5,6^.

At the end of the last century, the feasibility and application of titanium alloys in oil and gas development had studied worldwide, and the performance of titanium alloys OCTGs in oil and gas exploration conditions was evaluated in detail. It had been concluded that titanium alloy materials have significant advantages and application prospects in oil and gas exploration environments^5,7^. The United States, Japan and other countries had developed commercial products of titanium alloy tubular goods, such as tubing, casing, coiled tubing, drill pipe, drilling riser and catenary riser^8–11^. Schutz and Jackie verified the feasibility of using titanium alloy materials in high-temperature, high-pressure and high-corrosion environments through experimental methods since the 1990s, RMI Titanium Company developed titanium alloy tubing, casing, marine risers and other products with strength exceeding 1000 MPa, and these products have been successfully applied in multiple oil and gas fields in the United States, the Oryx Neptune project in the Gulf of Mexico and the thermal acid gas wells in the Mobile Bay oil field^12,13^. Titanium alloy materials such as Ti-6246, Ti–6Al–4V, Gr.29 and Beta-C developed by Chevron have been applied in ultrahigh-pressure high-temperature wells in the Gulf of Mexico^14^. In China, the CNPC Tubular Goods Research Institute (TGRI) conducted a comprehensive study of the feasibility of titanium alloy tubular goods for oil and gas development, it is proved that titanium alloy pipes had good application prospects under high-temperature and high-pressure exploration conditions. TGRI also defined the material selection map of titanium alloy OCTG used for oil and gas exploration and established related standards in China^6,15–17^. Tianjin Pipe Group Corporation, Panzhihua Iron and Steel Group and other companies have trial-produced titanium alloy tubing products^18–20^.

Due to the particularity of oil and gas resources in China, the service conditions underground have the characteristics of high temperature, high pressure and high corrosion, such as the Bozi-9 well of Tarim Oilfield in the western of China has reached a drilling depth of nearly 8000 m and bottom pressure of nearly 140 MPa^21^, this service condition require the higher requirements of titanium alloy tubing and casing, especially the collapse strength. However, the evaluation system and standards of OCTGs in the petroleum industry were established based on carbon steel, and titanium alloys are significantly different from steel in terms of elastic modulus and Poisson's ratio. There is no calculation model or standard for predicting the collapse strength of titanium alloy tubing and casing. The gap between the actual collapse strength of titanium alloy pipes and the calculation results of the collapse strength in API and ISO standard systems is still unknown. Bob Hargrave et al. conducted a physical collapse test on Ti–3Al–8V–6Cr–4Zr–4Mo (Beta-C) titanium alloy tubes^14^, and the results showed that the collapse strength of Beta-C titanium alloy pipes could only reach 90% of the theoretical calculation value according to the API 5C3 standard^22^. However, the collapse strength formula in the API 5C3 standard has many limitations, including that the calculation accuracy of this formula is not sufficient for small D/t ratio pipes, this formula cannot be applied to non-API OCTGs, and it cannot reflect the resistance induced by different manufacturing processes and heat treatments to the pipe materials. The Klever–Tamano formula recommended by the ISO RP 10400 standard^23^ has a calculation error of ± 35% for special OCTGs^24,25^, so it is difficult to use for the actual design and application of titanium alloy OCTGs. Because there is a lack of research on the collapse strength calculation and tests of titanium alloy OCTGs, the change rule of key parameters (such as strength, ovality, eccentricity and residual stress) on collapse strength of the tubing and casing is still unclear, and the difference between titanium alloy and steel pipe under the same specifications need to be further analyzed. In this paper, a new collapse strength prediction model, the strength collapse criterion model (SCM), was established for titanium alloy tubing and casing. The collapse strengths of titanium alloy tubing and casing were calculated by this mode and compared with the same specifications of steel pipes. The change in collapse strength with different key parameters was also investigated, and the calculated results were verified by the full-size physical collapse tests. This collapse strength calculation model will provide a technical reference and guidance for the design and application of titanium alloy tubing and casing.

## Theoretical model

**Figure 1:**
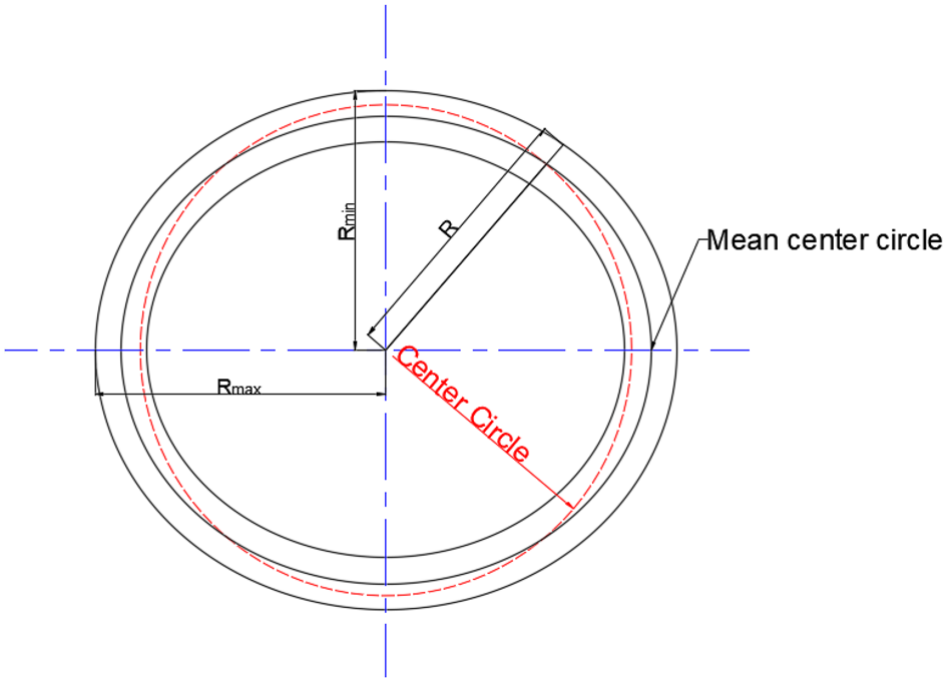
Actual tubing and casing cross-section.

The analysis and comparison of the full-scale collapse experiments of tubing and casing over the years shows that the collapse failure of the pipe occurs suddenly when the external pressure reaches the limit value. Because the shape of the pipe cannot reach an ideal circle, the stress of the noncircular pipes is not uniformly distributed in the circumferential direction, resulting in an additional bending moment effect. When the external pressure increases, the pipe yields at the place of maximum compressive hoop stress. When the yielding exceeds the limit value, the tubing and casing fail due to an insufficient bearing capacity, resulting in instability collapse, so the strength collapse criterion under external pressure is established as follows:

For the casing with initial ovality, assume the initial ovality of the center circle is $e_{0}$, the average radius is *R*, and the external pressure is *p*, as shown in Fig. 1. Assuming that the uniform wall thickness is *t*, the material is ideally elastoplastic, and the stress–strain relationship can be expressed as

1 $$ \sigma = \left\{ {\begin{array}{*{20}l} {E\varepsilon } \hfill & {\quad \varepsilon \le \varepsilon_{{\text{y}}} } \hfill \\ {\sigma (\varepsilon )} \hfill & {\quad \varepsilon > \varepsilon_{{\text{y}}} } \hfill \\ \end{array} } \right. $$

**Figure 2:**
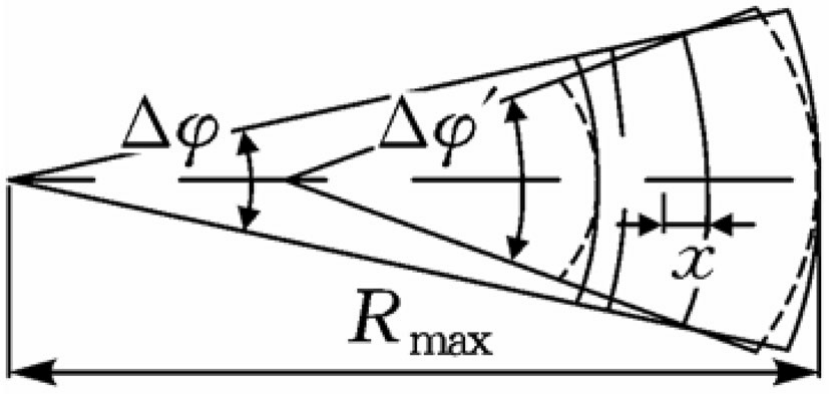
Deformed cell of the tubing and casing.

Taking the microelements from the long semi-axis of the cross-section of the casing (the thick solid line is the shape before deformation, and the dashed line is the shape after deformation), the angle before and after deformation is $\Delta \varphi$ and $\Delta \varphi ^{\prime}$, the radius of curvature of the central axis before and after deformation is $\rho$ and $\rho ^{\prime}$, respectively, as shown in Fig. 2, and the hoop strain generated by the bending moment at a distance x from the center circle is:

2 $$ \varepsilon_{\theta } = \frac{{\left( {\rho ^{\prime} + x} \right)\Delta \varphi ^{\prime} - \left( {\rho + x} \right)\Delta \varphi }}{{\left( {\rho + x} \right)\Delta \varphi }} $$

Assuming that when the pipe collapses, the material with a length of *s* from the inner surface at the long axis of the casing cross-section yields, and due to the symmetry of the casing with an elliptical cross-section, the radial shear stress is zero. For the convenience of calculation, it is assumed that the radial stress of the wall at the long semiaxis is linearly distributed without considering the plastic unloading effect. According to elastoplastic theory, the circumferential stress $\sigma_{\theta }$ and radial stress $\sigma_{r}$ are given by the following equations:

In the elastic zone

3 $$ \left( {s - t/2 \le x \le t} \right):\quad \sigma_{\theta } = E\varepsilon_{\theta } + \mu \sigma_{r} + \mu \sigma_{a} $$

In the plastic zone

4 $$ \left( {s - t/2 \le x \le t} \right):\quad \sigma_{\theta } = \frac{{\sigma_{r} + \sigma_{a} - \sqrt {4\sigma_{y}^{2} - 3\left( {\sigma_{r} - {\upsigma }_{{\text{a}}} } \right)^{2} } }}{2} $$

5 $$ \sigma_{r} \approx - \frac{P}{t}\left( {x + \frac{t}{2}} \right) $$

where $\sigma_{a}$ is the axial stress.

6 $$ \begin{aligned} {\text{At}}\;\;x & = s - \frac{t}{2}, \\ \sigma_{r} & = - p\frac{s}{t} \\ \end{aligned} $$

7 $$ \sigma_{\theta 2} = \frac{{ - \sigma_{r} + \sigma_{a} - \sqrt {4\sigma_{y}^{2} - 3\left( {\sigma_{r} - \sigma_{{\text{a}}} } \right)^{2} } }}{2} $$

8 $$ \varepsilon_{\theta 2} = \frac{1}{E}\left[ {\sigma_{\theta 2} - \mu \left( {\sigma_{a} - {\upsigma }_{{\text{r}}} } \right)} \right] $$

Substituting $x = s - \frac{t}{2}$ into Eq. (2), the following can be obtained:

9 $$ \varepsilon_{\theta } = \frac{{\left( {1 + \varepsilon_{\theta 2} } \right)\left( {\rho - \frac{t}{2} + S} \right)\left( {\rho^{\prime } + x} \right)}}{{\left( {\rho^{\prime } - \frac{t}{2} + S} \right)\left( {\rho + x} \right)}} - 1 $$

According to the balance between the force on the long semi-axis of the casing cross-section and the applied circumferential pressure, there is a similar relationship with the bending moment load effect:

10 $$ \begin{aligned} - N & = \mathop \smallint \limits_{ - t/2}^{t/2} \sigma_{\theta } \,dx \\ & = \mathop \smallint \limits_{ - t/2}^{{\frac{t}{ - 2} + s}} \frac{{\sigma_{r} + \sigma_{a} - \sqrt {4\sigma_{y}^{2} - 3\left( {\sigma_{r} - {\upsigma }_{{\text{a}}} } \right)^{2} } }}{2}dx + \mathop \smallint \limits_{{\frac{t}{ - 2} + s}}^{t/2} \left( {E\varepsilon_{\theta } + \mu \sigma_{r} + \mu \sigma_{a} } \right)dx \\ \end{aligned} $$

11 $$ \begin{aligned} M & = \mathop \smallint \limits_{ - t/2}^{t/2} \sigma_{\theta } x\,dx \\ & = \mathop \smallint \limits_{ - t/2}^{{\frac{t}{ - 2} + s}} \frac{{\sigma_{r} + \sigma_{a} - \sqrt {4\sigma_{y}^{2} - 3\left( {\sigma_{r} - {\upsigma }_{{\text{a}}} } \right)^{2} } }}{2}x\,dx + \mathop \smallint \limits_{{\frac{t}{ - 2} + s}}^{t/2} \left( {E\varepsilon_{\theta } + \mu \sigma_{r} + \mu \sigma_{a} } \right)x\,dx \\ \end{aligned} $$

where $N$ is the resultant axial force acting on the cross-section and *M* is the bending moment.

Taking a unit length of casing, according to the balance between the axial pressure and bending moment in the wall along the long semi-axis of the casing,

12 $$ N = - pR\left( {1 + e} \right) $$

13 $$ \begin{aligned} M & = M_{1} + M_{2} \\ & = - P\left( {\frac{{a^{2} bln\left( \frac{b}{a} \right)}}{{b^{2} - a^{2} }} - \frac{a}{2}} \right)R\left( {1 + e} \right) + pR^{2} \left( {1 + e} \right)e \\ \end{aligned} $$

where $a = R\left( {1 + e} \right) - t/2$, $b = R\left( {1 + e} \right) + t/2$, and $M_{1}$ is the additional bending moment of the section calculated according to the Lame formula of an ideal thick-walled cylinder.

For the ellipse $\rho = R\left( {1 + ecos\theta } \right)$, the relationship between the curve equation and the radius of curvature can be obtained from the relationship between the radius of curvature of the center circle at the long semi-axis before and after deformation as follows:

14 $$ \rho = R\left( {1 - 3e_{0} } \right),\quad \rho ^{\prime} = R\left( {1 - 3e} \right) $$

Combining Eqs. (6) to (14), the following equation can therefore be obtained:

15 $$ P = P\left( S \right) $$

According to the collapse criterion of the external pressure on the pipe^22,23^, as the external pressure gradually increases, the yield length *S* on the wall thickness section of the long semi-axis also increases until the external pressure reaches the maximum value. Therefore, the extreme value method can be used to obtain the instability and collapse pressure of the pipe. Therefore, the derivative of *S* gives the following equation:

16 $$ \frac{dP\left( S \right)}{{d S}} = 0 $$

Calculate *S* from Eq. (16), and substitute Eq. (14) to find the maximum external pressure load $P_{{{\text{max}}}}$ that the pipe can bear.

To simplify the calculation, the engineering calculation can take *S* = 0, which can ensure that the external collapse strength of the casing is within the safe range and that the calculation accuracy basically meets the requirements. Substituting *S* = 0 into Eq. (15), the new collapse strength prediction model can be obtained as following^26,27^:

17 $$ P = \frac{N}{{R\left( {1 + e} \right)}} = \frac{{ - 3\left( {e - e_{0} } \right)\left( {C + \rho D} \right)}}{{\left( {1 + e} \right)\left( {Re + B} \right)\left( {R - t/2 - 3eR} \right)}} $$

where

18 $$ e = \frac{{ - K + \sqrt {K^{2} - 4H\left( {D - t\mu \sigma_{\alpha } + tE} \right)} }}{{6R^{2} \left( {D - t\mu \sigma_{\alpha } + tE} \right)}}, $$

19 $$ A = F\left( {\frac{t\mu }{{2R}} - 1} \right)\left( {\rho - t/2} \right)\left( {t + \rho ln\frac{2\rho - t}{{2\rho + t}}} \right) $$

20 $$ B = \frac{{\mu t^{2} }}{12R} - \left( {\frac{{a^{2} bln\left( {b/a} \right)}}{{b^{2} - a^{2} }} - \frac{a}{2}} \right) $$

21 $$ C = t\left( {E + \sigma_{\theta 2} - \mu \sigma_{\alpha } } \right)\left( {\rho - t/2} \right), $$

22 $$ D = F\left( {\rho - t/2} \right)ln\frac{2\rho - t}{{2\rho + t}}, $$

23 $$ F = E + \sigma_{\theta 2} - \mu \sigma_{\alpha } , $$

24 $$ H = CB - 3RDBe_{0} - 3ARe_{0} + t\left( {\mu \sigma_{\alpha } - E} \right)\left( {R - t/2} \right)B, $$

25 $$ K = 3AR + CR - 3RDe_{0} R + 3RDB + t\left( {\mu \sigma_{\alpha } - E} \right)\left( {R - t/2} \right)R - 3RBt\left( {\mu \sigma_{\alpha } - E} \right) $$

## Experimental verification

**Figure 3:**
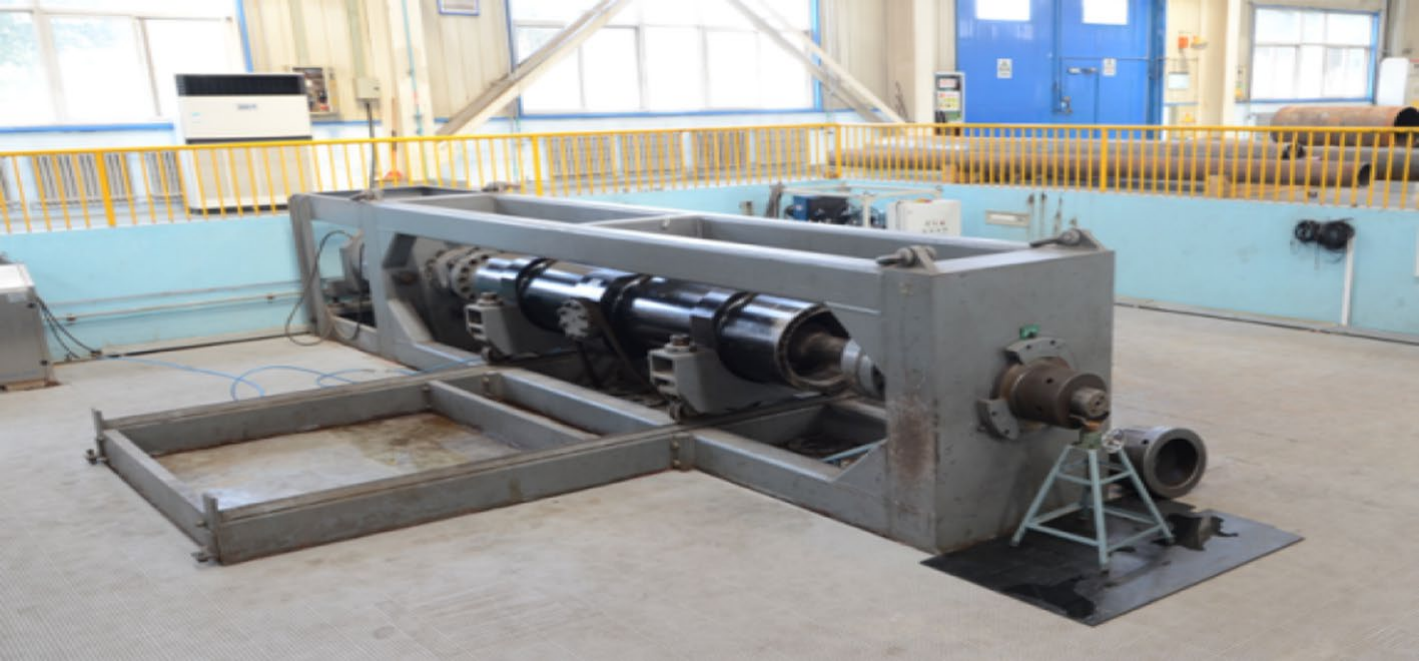
The full-scale collapse test device used in the test.

**Figure 4:**
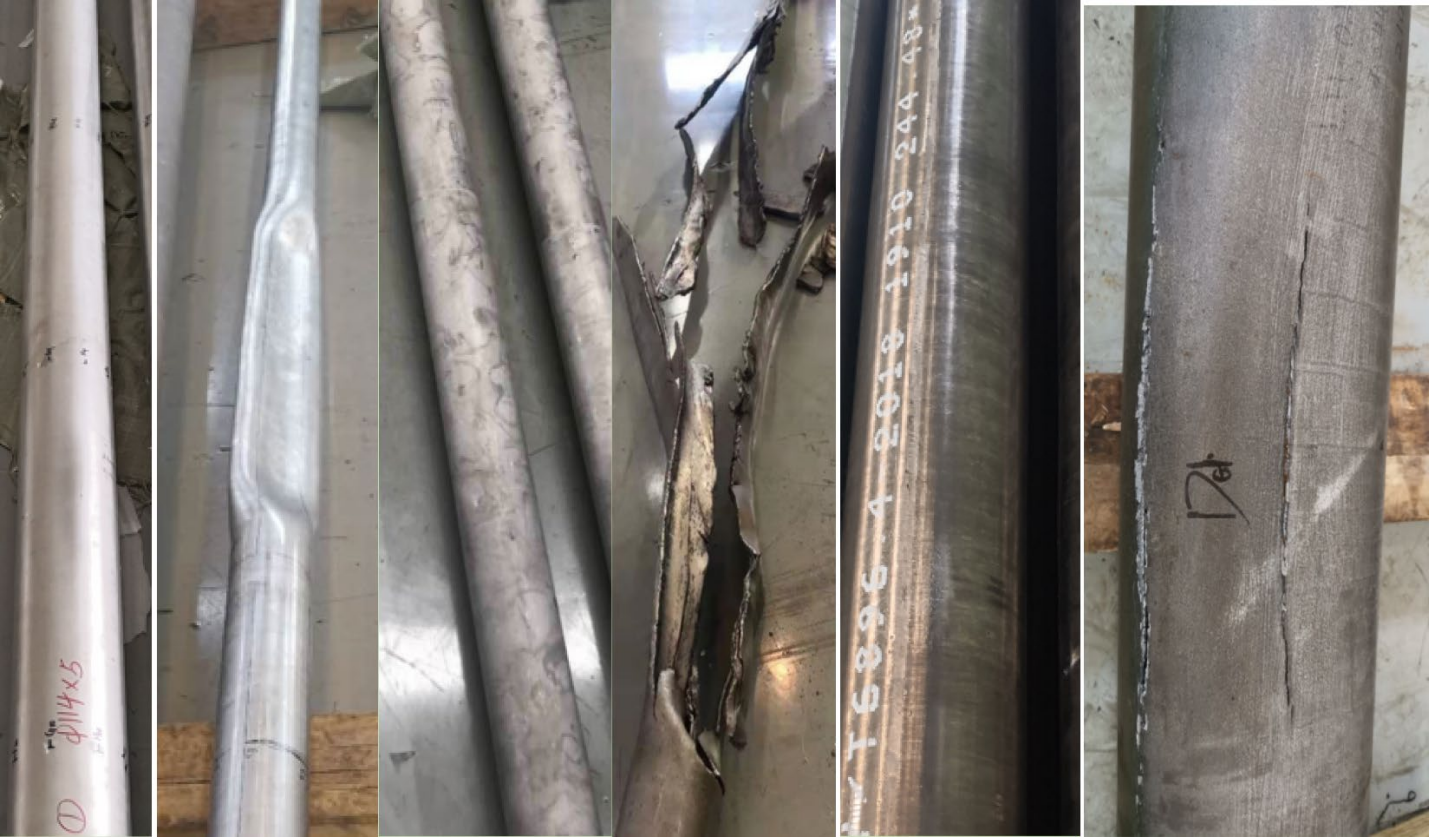
The morphology of samples before and after the tests.

To test the validity of the proposed calculation model, 25 different specifications of 758 MPa strength titanium alloy tubing and casing were selected for full-scale collapse testing. Before the tests, the external dimensions and wall thicknesses of all the pipes were measured to calculate the accurate D/t ratio. According to the standard API 5C5, the full-scale collapse test device in TGRI was used to uniformly apply external pressure to the tested titanium alloy tubing and casing until body instability/collapse. The full-scale collapse test device is shown in Fig. 3, and the testing was conducted at room temperature. The sample morphology before and after the test is shown in Fig. 4. These pictures were all captured form the actual test device and experiments in TGRI.

**Figure 5:**
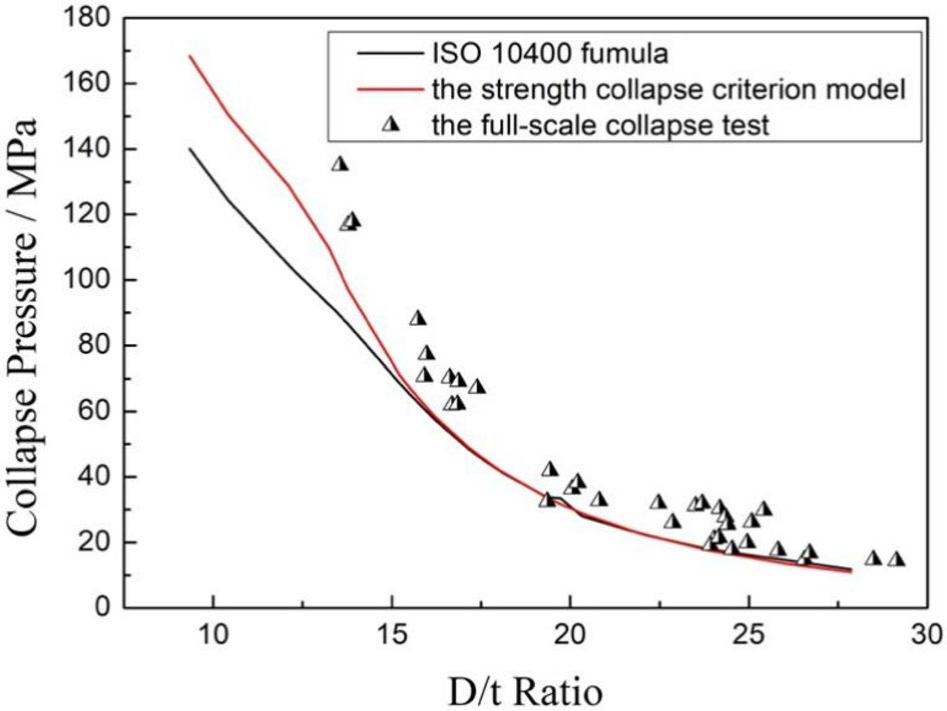
Comparison of the collapse strength of the tests and two calculation models.

Comparing the results of the full-scale collapse tests, the calculation values of the Klever–Tamano formula recommended by the ISO RP 10400 standard and the strength collapse criterion model, as shown in Fig. 5, it is revealed that the collapse strength calculated by the strength collapse criterion model and the Klever–Tamano formula were almost the same when the D/t ratio was greater than 15, and the full-scale collapse test results were close to the calculation results or slightly higher than the calculation results. When the D/t ratio was less than 15, the collapse strength calculated by the Klever–Tamano formula was much less than that of the strength collapse criterion model, and all the calculated results were smaller than the full-scale collapse test strength. The comparison results indicated that the strength collapse criterion model could predict a close approximation of actual collapse strength values for titanium alloy tubing and casing.

**Figure 6:**
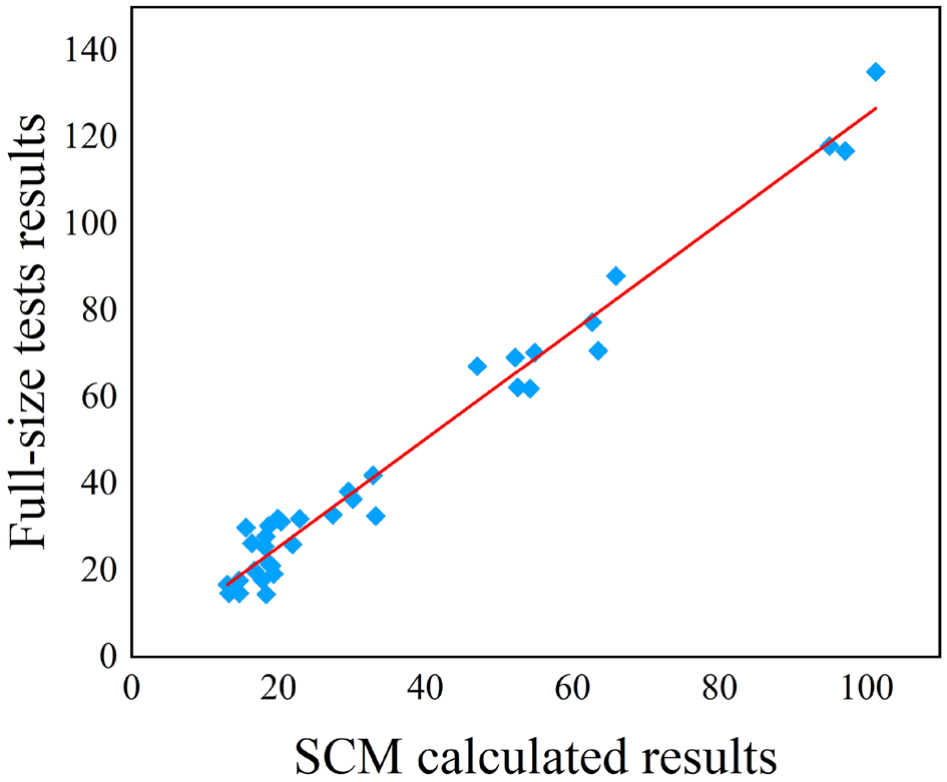
Comparison of experimental and calculated collapse strength of titanium alloy tubing and casing.

To verify the accuracy of the established strength collapse criterion model, the collapse strength of titanium alloy tubing and casing calculated by SCM model were compared with the results of the full-scale collapse tests. The comparison is shown in Fig. 9. It can be seen in the figure that the experimental values and predicted values are concentrated in the vicinity of the y = x line in the figure, and the correlation coefficient (R^2^) is 0.974, indicating that the absolute error value of the prediction of the collapse strength is less than 3%, and it has relative high prediction accuracy, which can provide a good prediction for the calculation of the collapse strength for titanium alloy tubing and casing (Fig. 6).

## Deep analysis and discussion based on SCM model

Due to the difference of equipment and processing in the preparation process of the tubing and casing, the actual tubing and casing shape parameters such as ovality, eccentricity, strength and residual stress will fluctuate within a certain range. The changes in these key parameters will significantly affect the collapse strength of the tubing and casing products. Previous full-scale collapse tests have proved that the strength collapse criterion (SCM) model can effectively predict collapse strength values for titanium alloy tubing and casing, so it is necessary to conduct an in-depth analysis to the influence of key parameters on the collapse strength of titanium alloy pipes compared with steel pipes based on the SCM model.

### Effect of ovality on collapse strength

**Figure 7:**
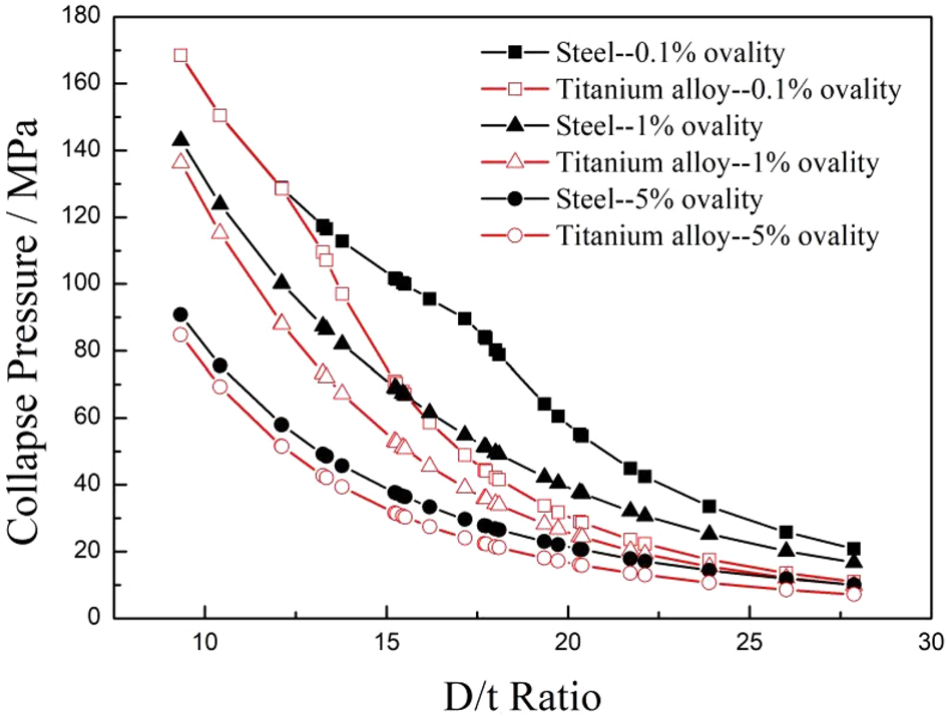
Comparison of the collapse strength of two different materials with various ovalities.

To investigate the influence of ovality on the collapse strength of tubing and casing, it is assumed that the calculated pipes are not affected by the axial load, the external diameter and wall thickness are nominal values from API Spec 5CT, there is no residual stress, the wall thickness is uniform, the yield strength is the nominal yield strength at the 758 MPa level, the elastic modulus and Poisson's ratio of the carbon steel material are 207 GPa and 0.33, respectively, and the elastic modulus and Poisson's ratio of the titanium alloy are 110 GPa and 0.3, respectively. 28 typical specifications of tubing and casings with an outer diameter of 73.02–339.72 mm were selected from API Spec 5CT standard for calculation, and the ovality of the calculated pipes was set at 0.1%, 1%, and 5%, respectively. The calculated collapse strengths of the titanium alloy and carbon steel pipes are shown in Fig. 7.

**Figure 8:**
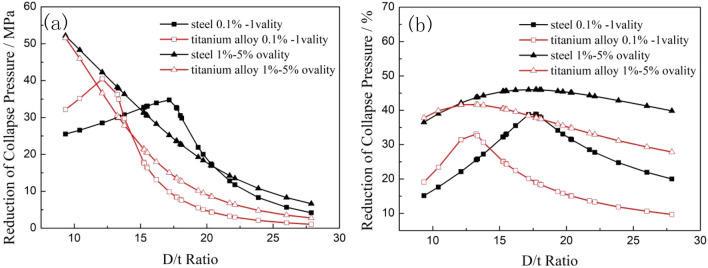
Comparisons of the reduction magnitudes (**a**) and percentages (**b**) of the collapse strength of two kinds of pipes with different ovalities.

Figure 7 shows that the collapse strengths of both the titanium alloy and carbon steel pipes decreased with increasing of D/t ratio, and the collapse strength of the titanium alloy pipes was significantly smaller than that of the carbon steel pipes under the same specification. When the ovality of the tubing and casing increased from 0.1 to 5%, the collapse strengths of the titanium alloy pipes and steel pipes under the same specification both decreased remarkably, however, with the increase of ovality, the change law of the collapse strength between the titanium alloy pipes and carbon steel pipes changed significantly. When the ovality increased from 0.1 to 1%, the reduction in the collapse strength of the titanium alloy pipes was higher than that of the carbon steel pipes within the low D/t ratio range, but this reduction was much less than that of the carbon steel pipes when the D/t ratio was greater than 14. The collapse strength reductions of the two material tubes showed the characteristics of first increasing and then decreasing with the increasing of D/t ratio. When the ovality further increased from 1 to 5%, the reductions in the collapse strengths of both materials dropped exponentially, and the reductions in the collapse strengths of the titanium alloy pipes were less than that of the carbon steel pipes for all D/t ratios, as shown in Fig. 8.

**Figure 9:**
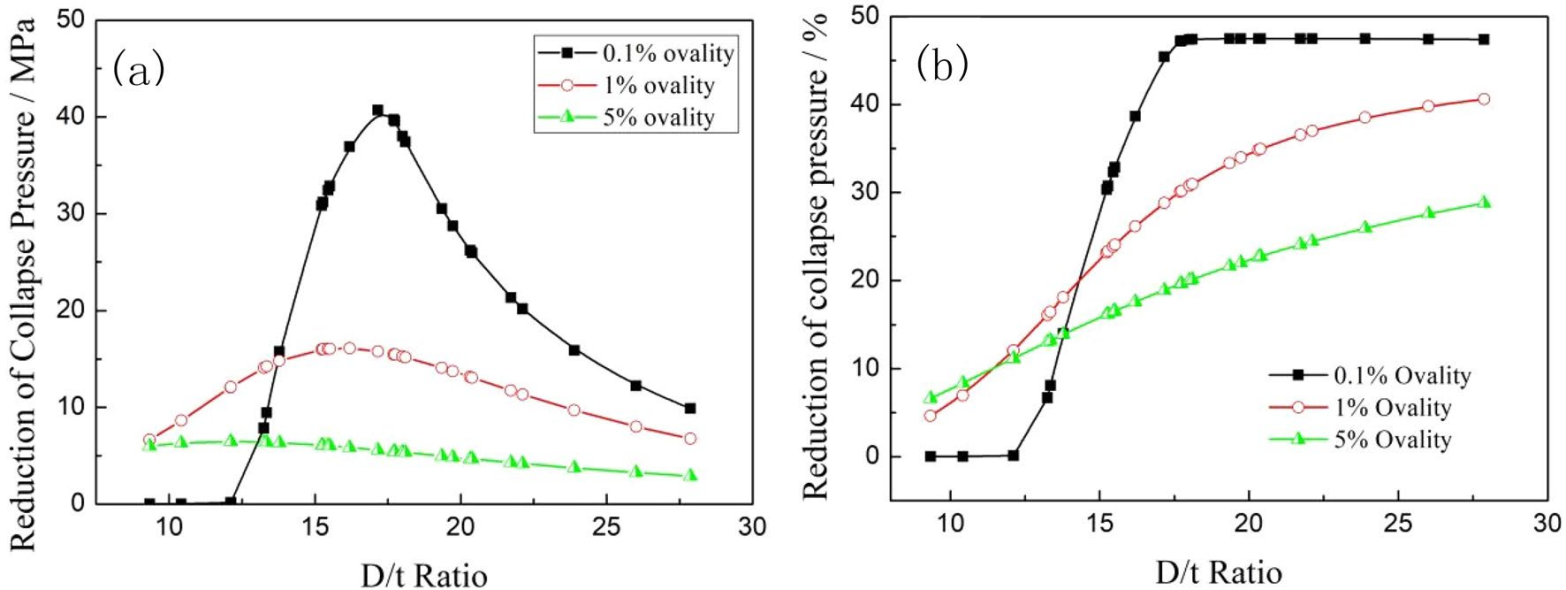
Comparison of the collapse pressure gaps (**a**) and reduction percentages (**b**) between carbon steel and titanium alloy with different ovalities.

The difference in the collapse strength (the collapse strength “gap”) and reduction percentage between carbon steel pipes and titanium alloy pipes with different ovalities are shown in Fig. 9. When the ovality was 0.1%, the collapse strength gap between the two types of pipes increased rapidly until a peak and then dropped quickly with the increase of D/t ratio. The maximum collapse strength gap (40.71 MPa) between the titanium alloy and carbon steel pipes occurred when the D/t ratio was 17.16. When the D/t ratio was less than 12.12, the collapse pressure gap between the two types of pipes was less than 0.2 MPa, and the reduction percentage was less than 1%, which means that plastic collapse occurred mainly in both materials pipes within this D/t ratio range. When the D/t ratio was greater than 17.7, the reduction percentage of the collapse strength tended to be stable at 47–48%, that can be explained that the collapse mode of the tubing and casing is elastic instability collapse when the D/t ratio is large, and the decrease in the elastic modulus from the titanium alloy to the carbon steel material is approximately 47%. The collapse strength gap between the titanium alloy pipes and carbon steel pipes significantly decreased with the increase of ovality, it is revealed that the influence of material differences on the collapse strength can be diminished by increasing the ovality.

### Effect of eccentricity on collapse strength

**Figure 10:**
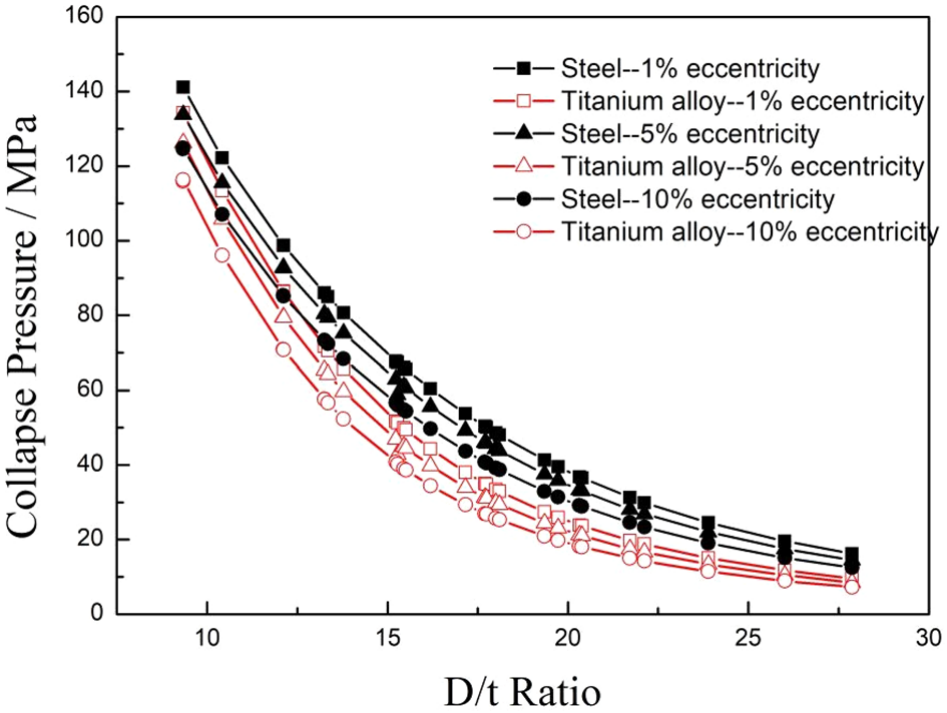
Comparison of the collapse strengths of pipes made of two different materials with different eccentricities.

In order to evaluate the effect of eccentricity on the collapse strength of titanium alloy and steel pipes, the eccentricity of the calculated pipes was assumed to be 1%, 5%, and 10%. All the calculation parameters were the same as the settings in the ovality analysis above except that the ovality of the pipe was set to 1%. The calculated collapse strength results of 28 typical specifications of titanium alloy and carbon steel pipes are shown in Fig. 10.

Figure 10 shows that the collapse strength of the titanium alloy pipes was smaller than that of the steel pipes for the same eccentricity and specification, and the collapse strength of both the titanium alloy and carbon steel pipes decreased with the increase of eccentricity, it is indicated that eccentricity had a negative effect on the collapse strength of OCTGs. The collapse strengths of both kinds of pipes dropped exponentially with the increase of the D/t ratio.

**Figure 11:**
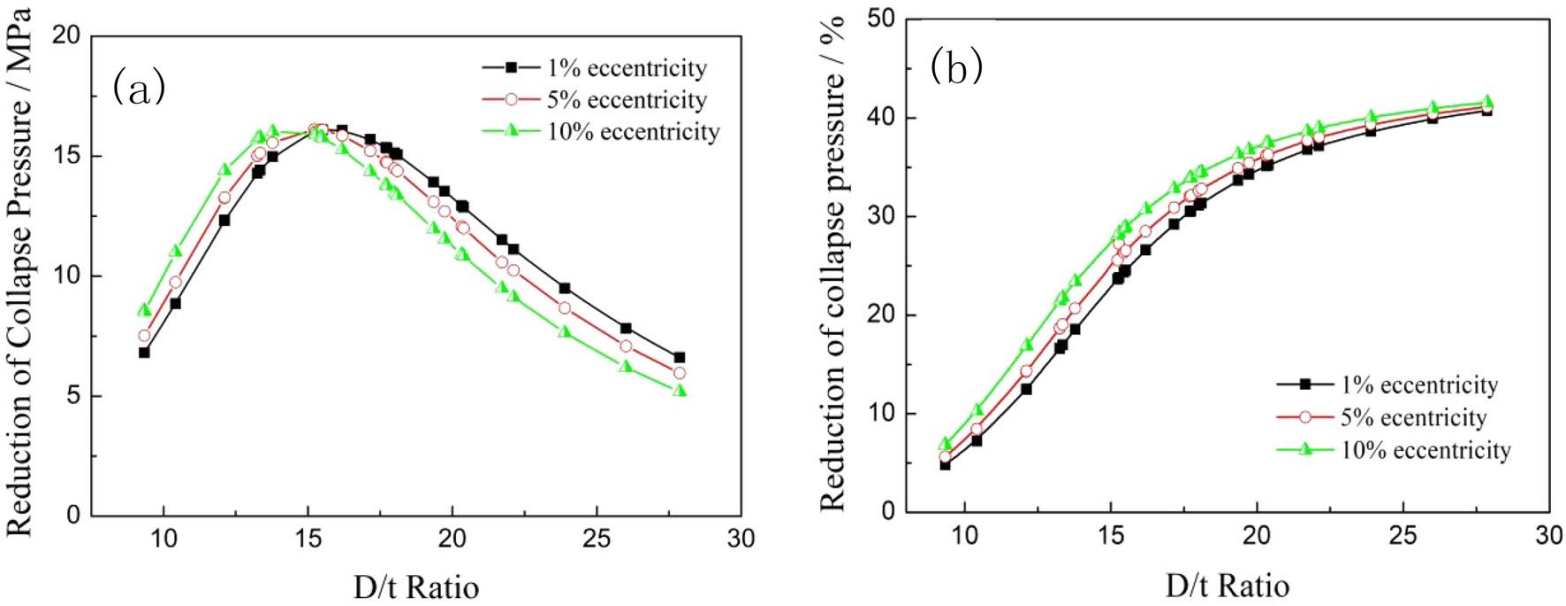
Comparison of collapse pressure gaps (**a**) and reduction percentages (**b**) between the steel and titanium alloy pipes with different eccentricities.

The collapse strength gap and reduction percentage between the steel pipes and titanium alloy pipes with different eccentricities are shown in Fig. 11. The collapse strength gap increased rapidly until a peak and then dropped quickly with the increase of the D/t ratio under all the eccentricity conditions, the maximum collapse strength gap between the titanium alloy and the steel pipes were maintained at approximately 16 MPa with different eccentricities, and the D/t ratio position where the maximum collapse strength gaps occurred decreased from 15.51 to 13.78 with the increase of eccentricity. When the D/t ratio was less than the value at which the maximum collapse strength gaps occurred, the reduction in the collapse strength of the two material pipes increased with the increase of eccentricity. On the contrary, the reduction in the collapse strength decreased with the increase of the D/t ratio at the large D/t ratio range. The reduction percentage of the collapse strength exponentially increased with the increase of the D/t ratio, which means that the influence of eccentricity on collapse strength was not as obvious as that of the D/t ratio, as shown in Fig. 11b.

### Effect of residual stress on collapse strength

**Figure 12:**
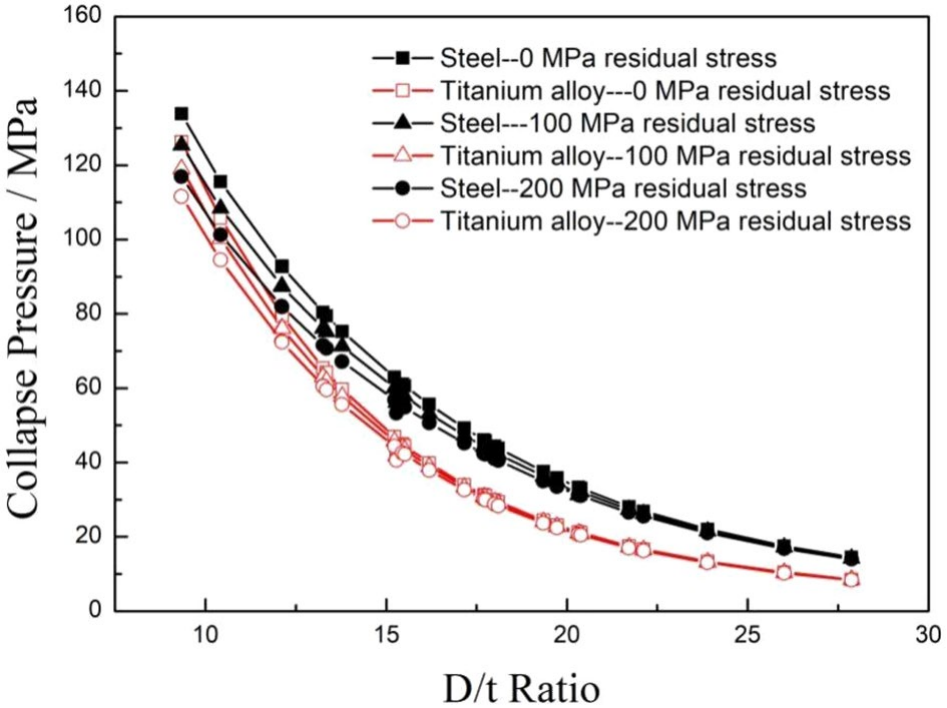
Comparison of the collapse strengths of the two types of pipes with different residual stresses.

The effect of residual stress on the collapse strength of titanium alloy pipes and steel pipes was evaluated by assuming the residual stress values of 0 MPa, 100 MPa and 200 MPa. The calculation parameters were the same as the settings in the eccentricity analysis above except that the eccentricity of the tubing and casing was set to 5%. The collapse strength comparison of the steel pipes and titanium alloy pipes with different residual stresses were calculated and is shown in Fig. 12. The collapse strengths of both the titanium alloy and carbon steel pipes decreased exponentially with the increase of the D/t ratio, and the collapse strengths of the titanium alloy pipes were significantly smaller than those of the steel pipes under the same calculation conditions. When the residual stress of the calculations increased from 0 to 200 MPa, the collapse resistances of both two material pipes slightly reduced in the low D/t ratio area, but there was almost no difference in the high D/t ratio area, it is indicated that the residual stress had only a minor effect on the collapse resistances of pipes with a high D/t ratio. Furthermore, the effect of the residual stress on the titanium alloy tubing and casing was smaller than that of the steel pipes.

**Figure 13:**
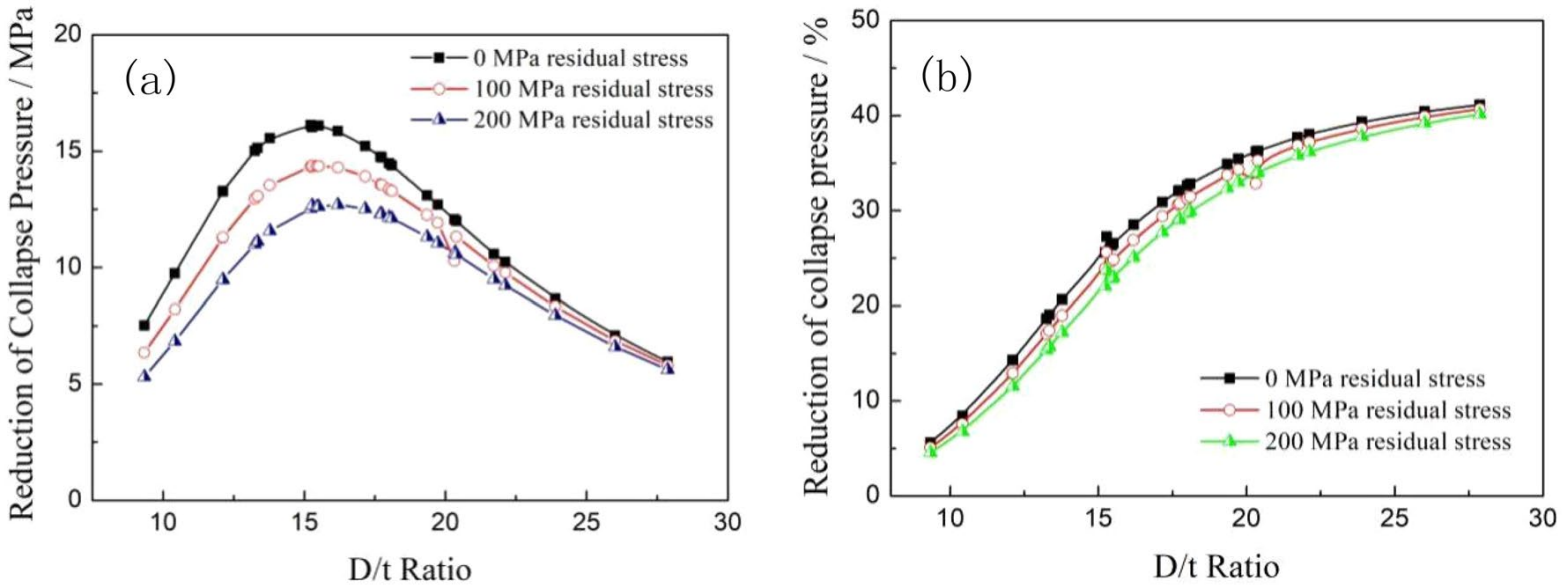
Comparison of collapse pressure gaps (**a**) and reduction percentages (**b**) between the carbon steel and titanium alloy pipes with different residual stresses.

Figure 13 shows the collapse strength gap and reduction percentage between the steel pipes and titanium alloy pipes with the increase of residual stress. The collapse strength gaps and the reduction percentages of collapse strength between the titanium alloy and carbon steel pipes decreased with increasing residual stress. The collapse strength gaps reached to a peak in the range of D/t ratios of 15–16 under all the residual stress conditions, and the maximum reduction values decreased with increasing residual stress. The reduction percentage of collapse strength between the titanium alloy and the steel pipes increased exponentially with the increase of the D/t ratio, as shown in Fig. 12b. It can be disclosed that the existence of residual stress can significantly reduce the collapse resistance of both titanium alloy and carbon steel pipes, but the degree of influence on the collapse strength of the titanium alloy tubing and casing decreased with the increase of residual stress.

### Effect of strength on collapse strength

**Figure 14:**
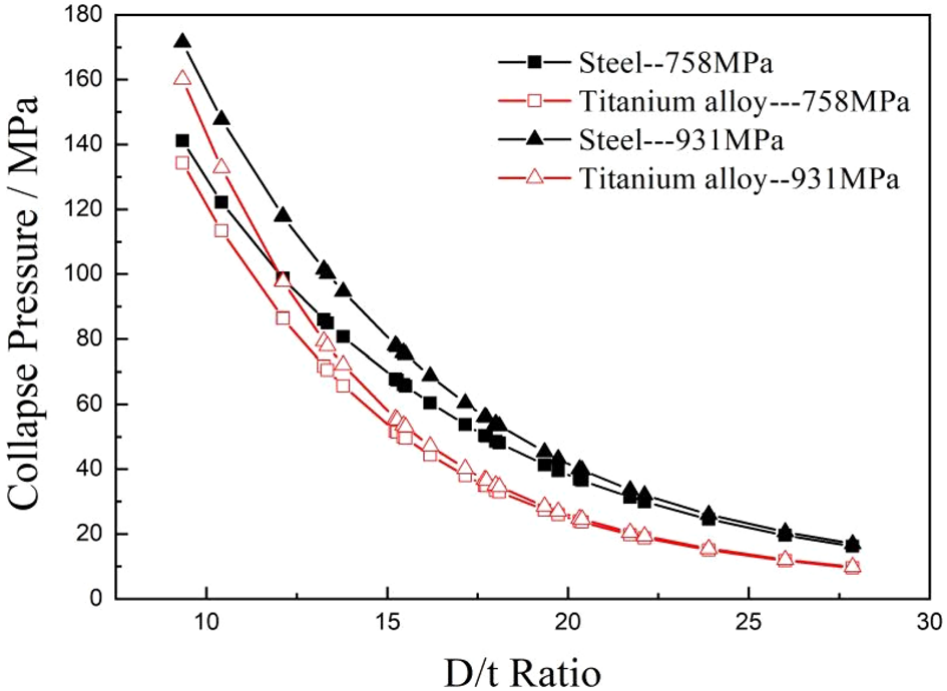
Comparison of the collapse strengths of two different types of pipes made of materials with yield strengths of 758 MPa and 931 MPa.

Titanium alloy pipes and steel pipes with 758 MPa and 931 MPa yield strengths were selected to calculate their collapse strengths. The ovality and eccentricity of the calculated pipes were set to 1% and 5%, and the residual stress of both pipes was set to 100 MPa. The other calculation parameters were the same as the settings in the analysis above. The calculation results shows that the collapse strengths of both two pipes can be significantly improved, and the effect of yield strength on the collapse strengths is more obvious in the low D/t ratio area. As the D/t ratio increases, the effect of yield strength on the collapse resistance of pipes gradually disappears, especially for titanium alloy pipes. No obvious changes were observed when the D/t ratio was greater than 20, as shown in Fig. 14.

**Figure 15:**
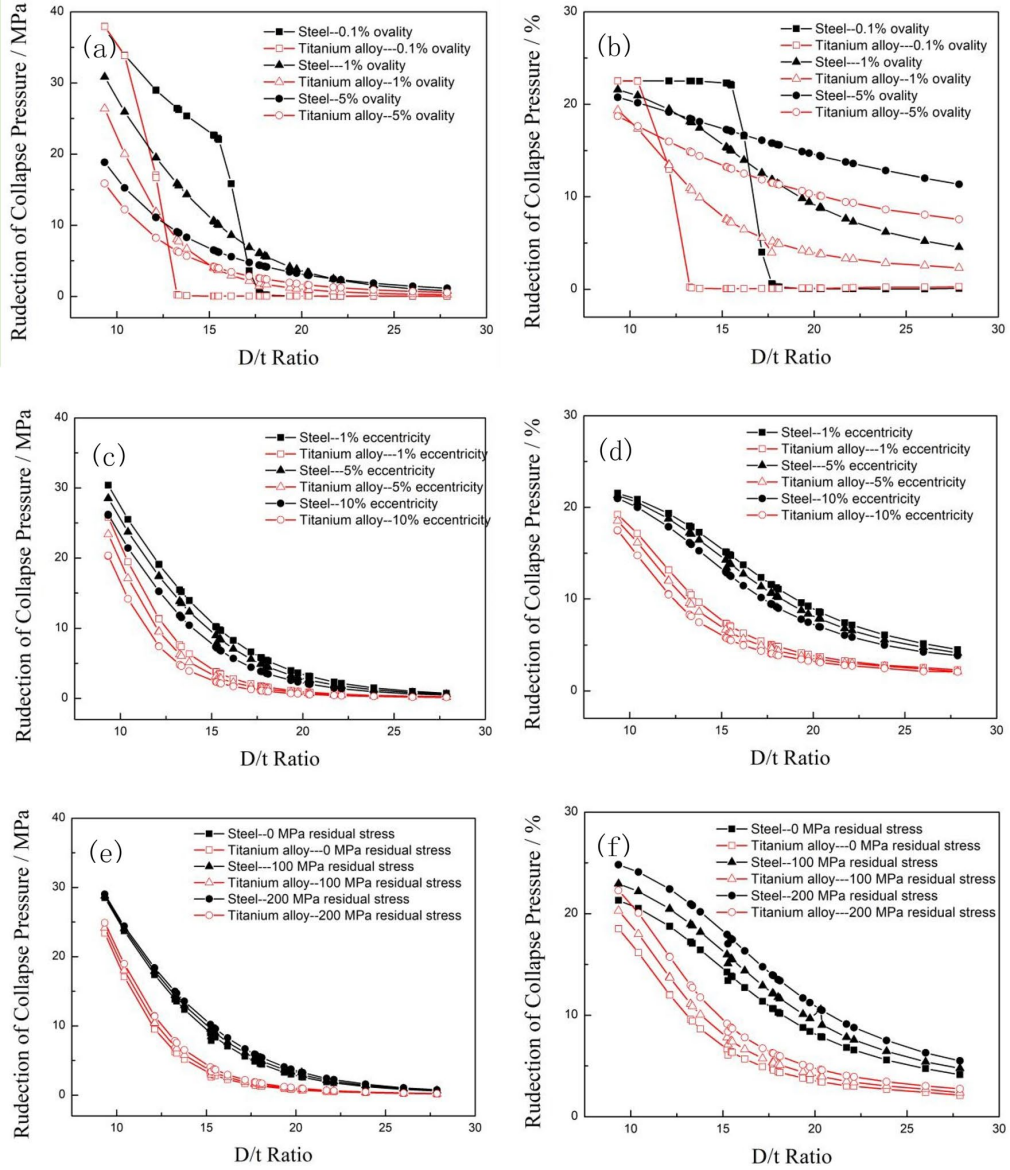
Comparison of collapse pressure gaps (**a**) and reduction percentages (**b**) between 758 and 931 MPa pipes with increasing D/t ratio and different key parameters.

The influence of collapse strength and other key parameters on the collapse resistance of two types of pipes were also investigated and shown in Fig. 15, the ovality had a great impact on the collapse strength reduction between the 758 and 931 MPa pipes, especially when the ovality was low, meanwhile, the effects of eccentricity and residual stress on the reduction of collapse resistance between the 758 and 931 MPa pipes were very small. When the D/t ratio is greater than 17, the collapse resistances of the 758 MPa and 931 MPa titanium alloy pipes were almost the same, indicating that elastic instability is the main influencing factor.

### Discussion

**Figure 16:**
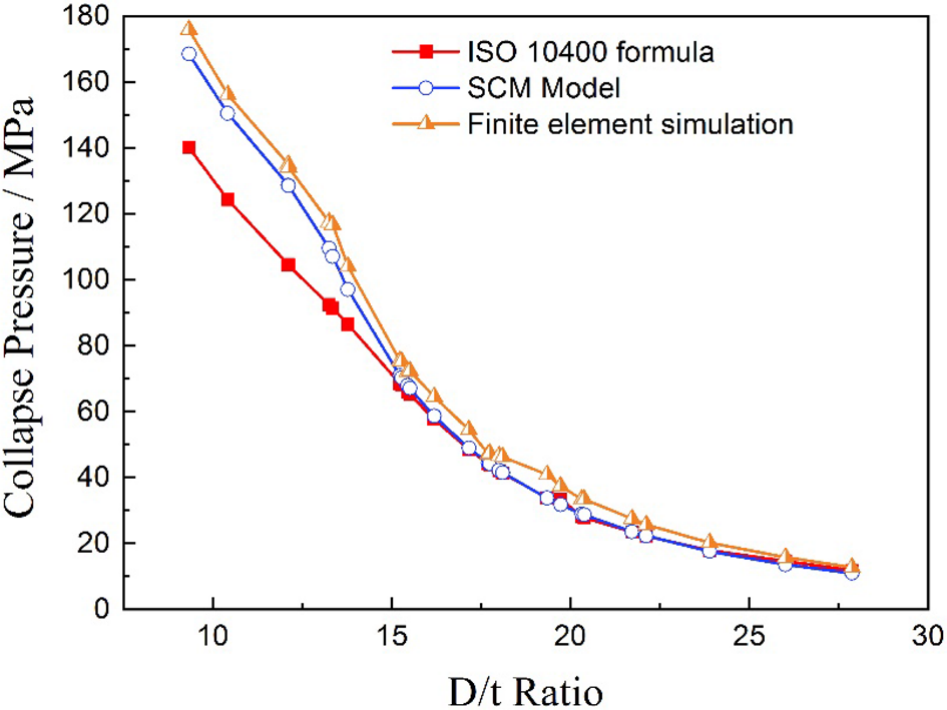
Comparison of collapse strength calculated by three methods.

The experimental verification tests showed that the strength collapse criterion model (SCM) which proposed in this paper can provide a good prediction for the calculation of the collapse strength for titanium alloy tubing and casing, Compared with the Klever–Tamano formula recommended by the ISO RP 10400 standard and FEA method used a lot^27,28^, the comparison of the calculation results calculated by three methods is shown in Fig. 16, it is can be seen that the calculation results of three prediction methods were almost the same when the D/t ratio was larger than 15, the collapse strength value calculated by the FEA method was highest among the three methods, and the result calculated by the SCM model was smaller when the D/t ratio was greater than 24, indicating that the SCM model predicts higher safety. When the D/t ratio was smaller than 15, the collapse strength value calculated by Klever–Tamano formula were much lower than the other two methods and the experimental results, indicating that the Klever–Tamano formula method has a large prediction error, resulting in waste of material properties and increased cost in actual use. The results of collapse strength predicted by the SCM model and the FEA method are much closer to the experimental values, and have a certain safety factor at the same time, which can be effectively used in design and check of the pipe string. Comparatively speaking, it can be concluded that the SCM model combines the advantages of the FEA method and the Klever–Tamano formula well, while avoiding the shortcomings of the two methods, and has good prediction accuracy in a wide range of pipe specifications, as shown in the Fig. 6, it can be effectively used to predict the collapse resistance of tubing and casing with various materials and specifications.

From the above in-deep analysis of key parameters, it also can be conducted that the ovality had the most significant negative effect on the collapse strength of titanium alloy tubing and casing, due to the ovality of pipe can greatly affect the radius of curvature of the center circle at the long semi-axis in Eq. (14), thus the ovality is the most important parameter for the collapse strength of OCTGs, the reductions of collapse strengths of both kinds of pipes dropped exponentially when the ovality increased from 1 to 5%. Meanwhile, it was worthy to note that the D/t ratio was another important key parameter for titanium alloy tubing and casing, in the part of collapse strength comparison between the full-scale tests and calculation models, there was remarkable difference when the D/t ratio is greater than 15 and less than 15. Moreover, the influences of the ovality, residual stress and strength on collapse strength of tubing and casing can be diminished when the D/t ratio is larger, it is because that when the D/t ratio is relatively large, the main collapse mechanism of the pipe under external pressure is elastic collapse, and the difference in elastic modulus between titanium alloy tubing and steel pipe is continuously reduced, the collapse strength properties of tubing and casing are mainly determined by the original geometric size and deformation of the pipe body. Therefore, the SCM model established in this paper can more accurately calculate the collapse resistance of pipes without the influence of elastic modulus. The model has higher prediction accuracy in the elastic collapse section, and can also be widely used in anti-collapse performance prediction and evaluation of many other materials.

## Conclusions

In this study, a new collapse strength calculation model for tubing and casing based on the strength collapse criterion model (SCM) was proposed. Full-scale collapse tests were carried out to verify the reliability of the calculation model, and the collapse strengths of titanium alloy pipes and steel pipes with different key parameters were calculated and compared. The following conclusions can be drawn from this work:

1. A new collapse strength prediction model, the strength collapse criterion model (SCM), was established for titanium alloy tubing and casing. This model assumes that the circumferential stress of a non-ideal circular pipe is not uniformly distributed along the circumference under an external pressure, and an additional bending moment effect exists. When the external pressure increases, the pipe yields at the maximum compressive hoop stress and collapses due to an insufficient bearing capacity, resulting in strength or instability collapse.
2. 35 different specifications of 758 MPa strength titanium alloy tubing and casing were selected for full-scale collapse tests to verify the reliability of this SCM model. All the results of the full-scale collapse test were slightly above or close to the calculated results, indicative of a good prediction accuracy for titanium alloy tubing and casing.
3. The in-depth analysis of the influence of key parameters on collapse strength based on the SCM model showed that the ovality had a significant negative effect on collapse strength of pipe, and the influence of material differences on collapse strength can be diminished with the increase of the ovality, the influences of the eccentricity, residual stress and strength on collapse strength were much smaller than that of ovality.
4. In particular, the influences of residual stress and strength can be ignored when the D/t ratio is greater than 20. The collapse strengths of both the titanium alloy and the steel pipes decreased with the increase of the D/t ratio, and the collapse strengths of the titanium alloy pipes were smaller than that of the steel pipes under the same specification due to the low elastic modulus of titanium alloy.
